# ﻿Diversity of spiders in Daba Mountain National Nature Reserve, Chongqing, China (I), three new *Cicurina* species of Cicurinidae (Araneae)

**DOI:** 10.3897/zookeys.1219.137354

**Published:** 2024-11-26

**Authors:** Lu-Yu Wang, Yan-Nan Mu, Ling-Xiang Yang, Zhi-Sheng Zhang

**Affiliations:** 1 Key Laboratory of Eco-environments in Three Gorges Reservoir Region (Ministry of Education), School of Life Sciences, Southwest University, Chongqing 400715, China Southwest University Chongqing China; 2 Chongqing Urban Ecosystem Positioning Observation and Research Station, Chongqing 400036, China Chongqing Urban Ecosystem Positioning Observation and Research Station Chongqing China; 3 Management Center of Daba Mountain National Nature Reserve, Chongqing 405909, China Management Center of Daba Mountain National Nature Reserve Chongqing China

**Keywords:** Morphology, new species, spider, taxonomy

## Abstract

Three new species of the genus *Cicurina* Menge, 1871 are described from Daba Mountain National Nature Reserve, Chongqing, China: *C.chengkou***sp. nov.** (♂♀), *C.dabashan***sp. nov.** (♂♀) and *C.longihamata***sp. nov.** (♂♀). Morphological descriptions, photos and illustrations of copulatory organs are provided.

## ﻿Introduction

The Daba Mountain National Nature Reserve is located in Chengkou County in the southern Dabashan Mountains, southwestern China. Its eastern and northern sides border Shaanxi Province. It is adjacent to Wuxi County and Kaizhou District of Chongqing Municipality in the south and is connected to Sichuan Province in the west. It is a transitional zone from the Qinling Mountains to southern China, extending from 31°37'27" to 32°12'15"N and 108°27'07" to 109°16'40"E. In the Daba Mountain National Nature Reserve, there are numerous valleys crisscrossing. The heterogeneous habitats create conditions for the independent evolution of species and have given birth to many endemic species such as ferns and flowering plants, including *Botrychiumsutchuenense*, *Aspleniumhumistratum*, *Primulafagosa*, and the beetle *Neobisniuschengkouensis* (Deng 2015).

A comprehensive survey of the biological resource background of Chongqing Daba Mountain National Nature Reserve was performed in 2011. In total, 181 macro fungi, 3572 tracheophyte, 884 insects, 68 mammalian, 233 avian, 24 reptilian, 25 amphibian and 44 fish species were recorded (Deng 2015). However, at present, only 13 species of spiders have been recorded in the Reserve: *Amaurobiusspinatus* Zhang, Wang & Zhang, 2018 (Amaurobiidae), *Chrosiothespengqi* Lin & Li, 2024, *Coscinidahantao* Lin & Li, 2024, *Mallinellazhoushengboi* Lin & Li, 2024, *Onomastuszhuwu* Lin & Li, 2024, *Orchestinaxiebao* Lin & Li, 2024, *Otacilialubrica* Mu & Zhang, 2021, *O.pyriformis* Fu, Zhang & Zhang, 2016, *O.wuli* Mu & Zhang, 2021, *Phricotelusyangxiong* Lin & Li, 2024, *Synagelideshuangxin* Lin & Li, 2024, *Tekellinahaosiwen* Lin & Li, 2024 and *Yaginumenaxuanzan* Lin & Li, 2024 (Theridiidae) (Fu et al. 2016; Zhang et al. 2018; Mu and Zhang 2021; Lin et al. 2024). The diversity of spiders is clearly underestimated.

*Cicurina* is the largest genus in the spider family Cicurinidae, with 144 species currently known worldwide. Most of them (114) are recorded from North America and a few (28) are found in Asia (Lin et al. 2023; WSC 2024). At present, there are 20 *Cicurina* species found in China (Li and Wang 2017; Wang et al. 2019; Liao et al. 2022).

This is the first study detailing the spider diversity of Daba Mountain National Nature Reserve. Three new species assigned to *Cicurina* Menge, 1871 are described: *C.chengkou* sp. nov., *C.dabashan* sp. nov. and *C.longihamata* sp. nov.

## ﻿Materials and methods

All specimens were preserved in 75% ethanol and were examined, illustrated, photographed and measured using a Leica M205A stereomicroscope equipped with a drawing tube, a Leica DFC450 camera and LAS software (ver. 4.6). Male pedipalps and epigynes were examined and illustrated after they were dissected. Epigynes were cleared immersing them in pancreatin (Álvarez-Padilla and Hormiga 2007). Eye sizes were measured as the maximum dorsal diameter. Leg measurements are shown as: total length (femur, patella and tibia, metatarsus, tarsus). All measurements are in millimetres. Specimens examined here are deposited in the Collection of Spiders, School of Life Sciences, Southwest University, Chongqing, China (SWUC).

Abbreviations used in the text: **ALE**, anterior lateral eye; **AME**, anterior median eye; **MOA**, median ocular area; **PLE**, posterior lateral eye; **PME**, posterior median eye.

## ﻿Taxonomy

**Family Cicurinidae Kishida, 1955**

**Genus *Cicurina* Menge, 1871**

### ﻿ Cicurina chengkou sp. nov.

Taxon classificationAnimaliaAraneaeHahniidae

0AC85C5B-5532-5559-AEC0-125FF0BD9C6E

https://zoobank.org/B8AE2F95-84BB-4A4F-954B-6DF16B89869E

**Figure 1.**
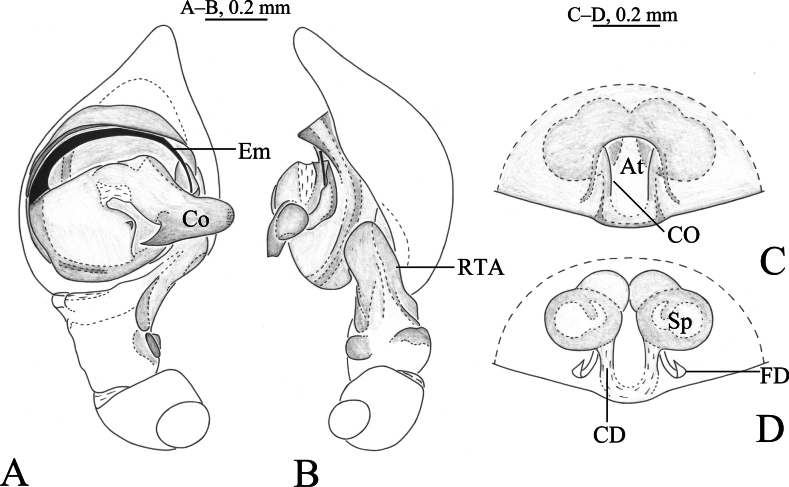
*Cicurinachengkou* sp. nov. holotype male (**A, B**) and paratype female (**C, D**) **A** left male palp, ventral view **B** same, retrolateral view **C** epigyne, ventral view **D** vulva, dorsal view. Abbreviations: At = atrium; CD = copulatory duct; CO = copulatory opening; Co = conductor; Em = embolus; FD = fertilization duct; RTA = retrolateral tibial apophysis; Sp = spermathecae.

**Figure 2.**
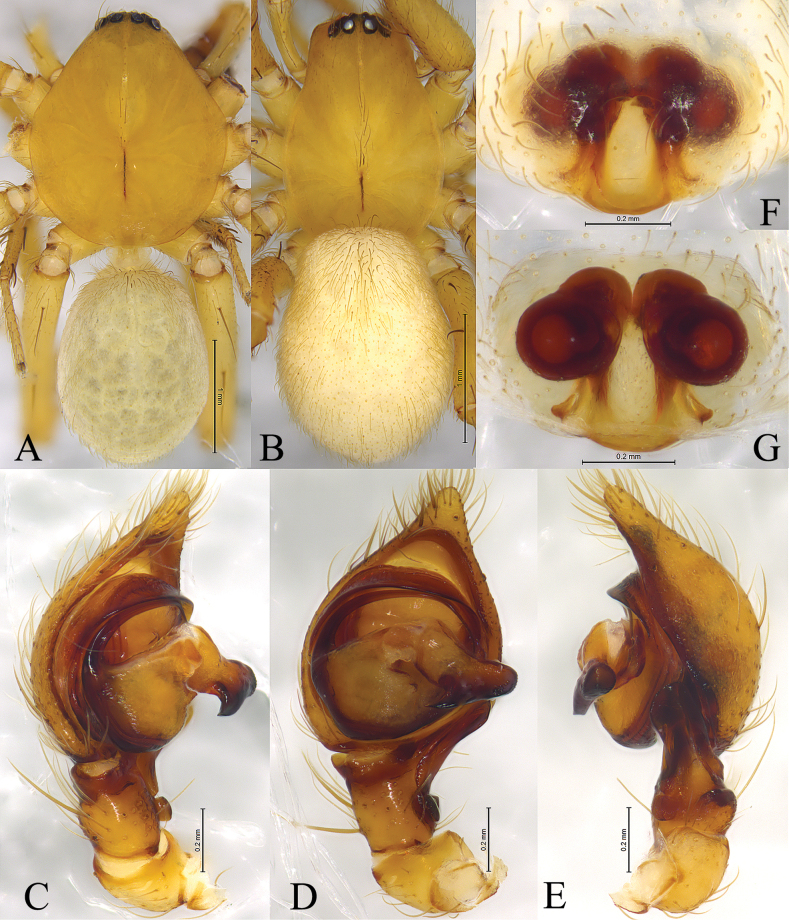
*Cicurinachengkou* sp. nov. holotype male (**A, C–E**) and paratype female (**B, F, G**) **A** male habitus, dorsal view **B** female habitus, dorsal view **C** left male palp, prolateral view **D** same, ventral view **E** same, retrolateral view **F** epigyne, ventral view **G** vulva, dorsal view.

Figs 1 , 2 , 7 Vernacular name: 城口洞叶蛛

#### Type material.

***Holotype*** • ♂ (SWUC-T-CI-10-01), **China**, Chongqing City, Chengkou County, Longtian Town, Wuli Village, Daba Mountain National Nature Reserve, 32°03.836'N, 108°40.238'E, elev. 1275 m, 21 March 2013, X.K. Jiang and X.W. Meng leg. ***Paratype*** • 1♀ (SWUC-T-CI-10-02), with same data as for holotype (SWUC).

#### Etymology.

The specific name is derived from the type locality.

#### Diagnosis.

**Figure 3.**
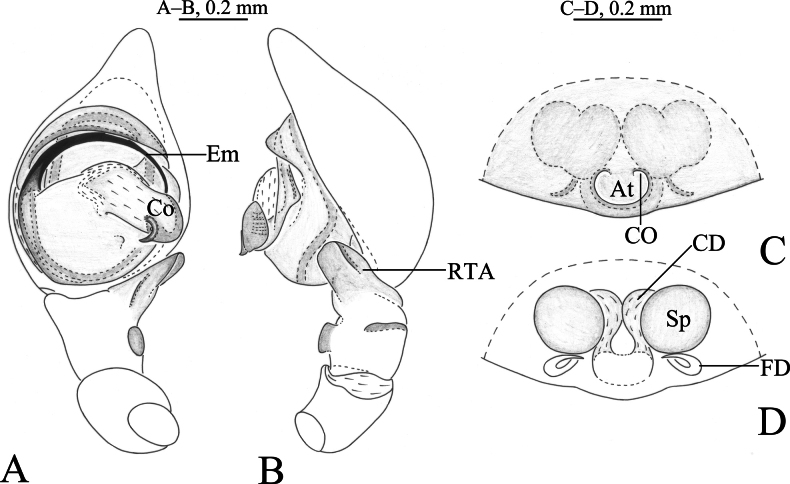
*Cicurinadabashan* sp. nov. holotype male (**A, B**) and paratype female (**C, D**) **A** left male palp, ventral view **B** same, retrolateral view **C** epigyne, ventral view **D** vulva, dorsal view. Abbreviations: At = atrium; CD = copulatory duct; CO = copulatory opening; Co = conductor; Em = embolus; FD = fertilization duct; RTA = retrolateral tibial apophysis; Sp = spermathecae.

**Figure 4.**
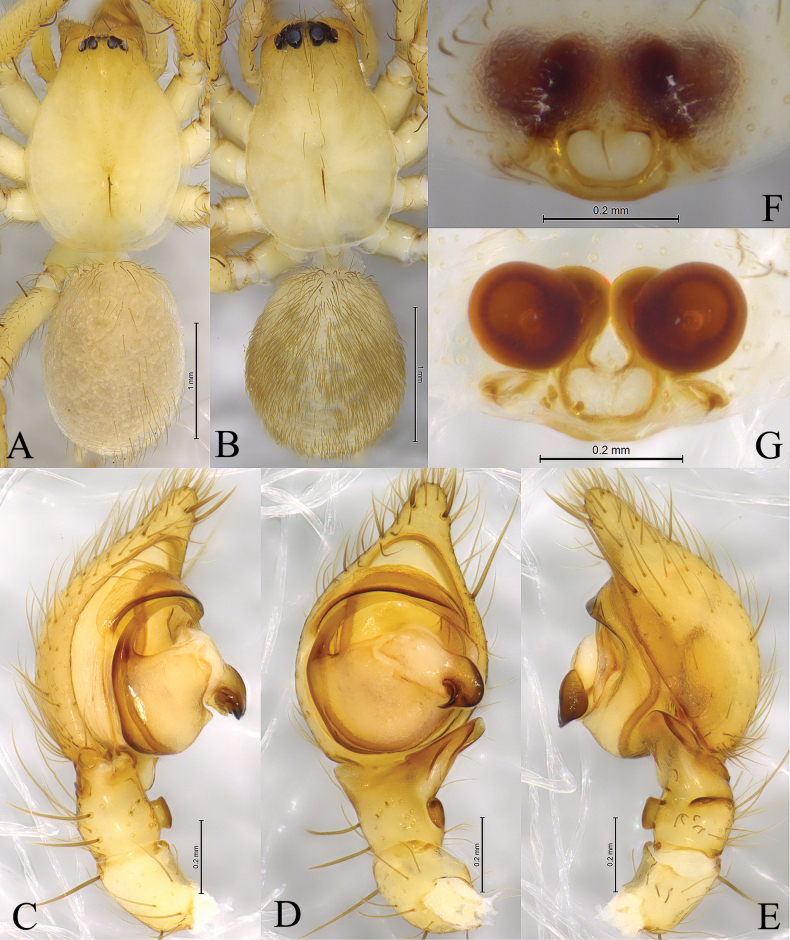
*Cicurinadabashan* sp. nov. holotype male (**A, C–E**) and paratype female (B, F, G) **A** male habitus, dorsal view **B** female habitus, dorsal view **C** left male palp, prolateral view **D** same, ventral view **E** same, retrolateral view **F** epigyne, ventral view **G** vulva, dorsal view.

The new species is similar to *C.dabashan* sp. nov. (Figs 3, 4) in having wide and short retrolateral tibial apophysis, long and strong embolus, strong and beak-like conductor, posteriorly located epigynal atrium and ball-shaped spermathecae, but differs from the latter by the long RTA (Figs 1B, 2Е) (vs. short in *C.dabashan* sp. nov.), the apical part of conductor as long as the width of conductor base (Figs 1A, B, 2C–E) (vs. about half the length of conductor base in *C.dabashan* sp. nov.) and the oblong atrium (Figs 1C, 2F) (vs. somewhat oval in *C.dabashan* sp. nov.), the short copulatory duct (Figs 1D, 2D) (vs. long in *C.dabashan* sp. nov.).

#### Description.

**Male holotype** (Fig. 2A) total length 4.02. Carapace 2.09 long, 1.75 wide; opisthosoma 1.78 long, 1.35 wide. Eye sizes and interdistances: AME 0.06, ALE 0.12, PME 0.10, PLE, 0.11; AME–AME 0.05, AME–ALE 0.04, PME–PME 0.09, PME–PLE 0.07, ALE–PLE 0.04. MOA 0.23 long, anterior width 0.18, posterior width 0.30. Clypeus height 0.21. Chelicerae with 3 promarginal and 8 retromarginal teeth. Leg measurements: I 5.74 (1.63, 1.98, 1.27, 0.86); II 5.32 (1.55, 1.80, 1.16, 0.81); III 4.78 (1.38, 1.54, 1.13, 0.73); IV 6.23 (1.63, 2.06, 1.62, 0.92). Leg formula: 4123.

***Palp*** (Figs 1A, B, 2C–E). Femur long, two times longer than cymbium, without modified. Tibia slightly longer than patella. Retrolateral tibial apophysis wide, with a single fold and rounded apex. Base of retrolateral tibial apophysis with two small apophyses, extending ventrally and dorsally. Bulb circular, tegulum semicircular, with a distinct process at middle part in prolateral and ventral view. Sperm duct obvious. Embolus strong, originating at approximately 9-o’clock position, anterior part resting in the long groove of conductor. Conductor strong, with a sharp end.

**Female paratype** (Fig. 2B) total length 3.51. Carapace 1.76 long, 1.23 wide; opisthosoma 1.86 long, 1.38 wide. Eye sizes and interdistances: AME 0.05, ALE 0.10, PME 0.08, PLE, 0.10; AME–AME 0.05, AME–ALE 0.02, PME–PME 0.08, PME–PLE 0.06, ALE–PLE 0.03. MOA 0.22 long, anterior width 0.15, posterior width 0.27. Clypeus height 0.14. Leg measurements: I 4.67 (1.39, 1.65, 0.95, 0.68); II 4.28 (1.23, 1.44, 0.94, 0.67); III 3.91 (1.15, 1.31, 0.93, 0.52); IV 5.26 (1.48, 1.76, 1.30, 0.72). Leg formula: 4123.

***Epigyne*** (Figs 1C, D, 2F, G). Atrium oval. Copulatory openings located lateral of atrium. Copulatory ducts straight and short, about half length of spermathecae diameter. Spermathecae kidney shaped. Fertilization ducts hook-like.

#### Distribution.

Known only from the type locality (Fig. 7).

### ﻿ Cicurina dabashan sp. nov.

Taxon classificationAnimaliaAraneaeHahniidae

6809F641-D75E-5C74-8B05-A3C072956946

https://zoobank.org/C5223A06-8D90-43EE-A991-385DC9EE7F65

Figs 3 , 4 , 7 Vernacular name: 大巴山洞叶蛛

#### Type material.

***Holotype*** • ♂ (SWUC-T-CI-11-01), **China**, Chongqing City, Chengkou County, Longtian Township, Wuli Village, Daba Mountain National Nature Reserve, 32°03.614'N, 108°40.316'E, elev. 1215 m, 16 September 2012, L.Y. Wang and X.K. Jiang leg. ***Paratypes*** • 1♀ (SWUC-T-CI-11-02), Wuli Village, Daba Mountain National Nature Reserve, 32°04.590'N, 108°39.058'E, elev. 1417 m, 17 September 2012, L.Y. Wang and X.K. Jiang leg. • 1♀ (SWUC-T-CI-11-03), Wuli Village, Daba Mountain National Nature Reserve, 32°04.432'N, 108°39.225'E, elev. 1353 m, 21 March 2013, X.K. Jiang and X.W. Meng leg.

#### Etymology.

The specific name is derived from the type locality (Dabashan = Daba Mountain).

#### Diagnosis.

The new species is similar to *C.lichuanensis* Wang, Zhou & Peng, 2019 (Wang, Zhou and Peng 2019: 354, figs 5A–D, 6A–G) in having similarly shaped retrolateral tibial apophysis, long and strong embolus, posteriorly located epigynal atrium and ball-shaped spermathecae, but differs from the latter by the tegulum with a small process (Figs 3A, 4C, D) (vs. without process), the conductor with a short, sclerotic and sharp end (Figs 3A, B, 4C–E) (vs. with a long and blunt end in *C.lichuanensis*) and the anterior edge of atrium unconnected (Figs 3C, 4F) (vs. connect in *C.lichuanensis*).

**Figure 5.**
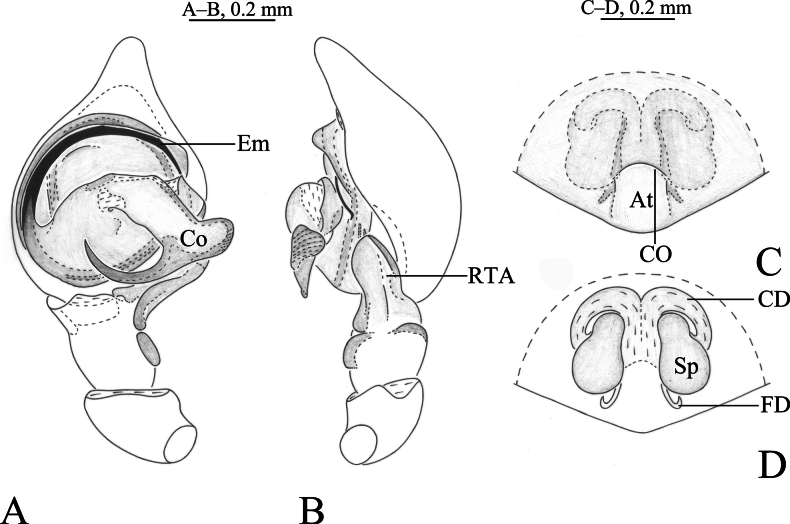
*Cicurinalongihamata* sp. nov. holotype male (**A, B**) and paratype female (**C, D**) **A** left male palp, ventral view **B** same, retrolateral view **C** epigyne, ventral view **D** vulva, dorsal view. Abbreviations: At = atrium; CD = copulatory duct; CO = copulatory opening; Co = conductor; Em = embolus; FD = fertilization duct; RTA = retrolateral tibial apophysis; Sp = spermathecae.

**Figure 6.**
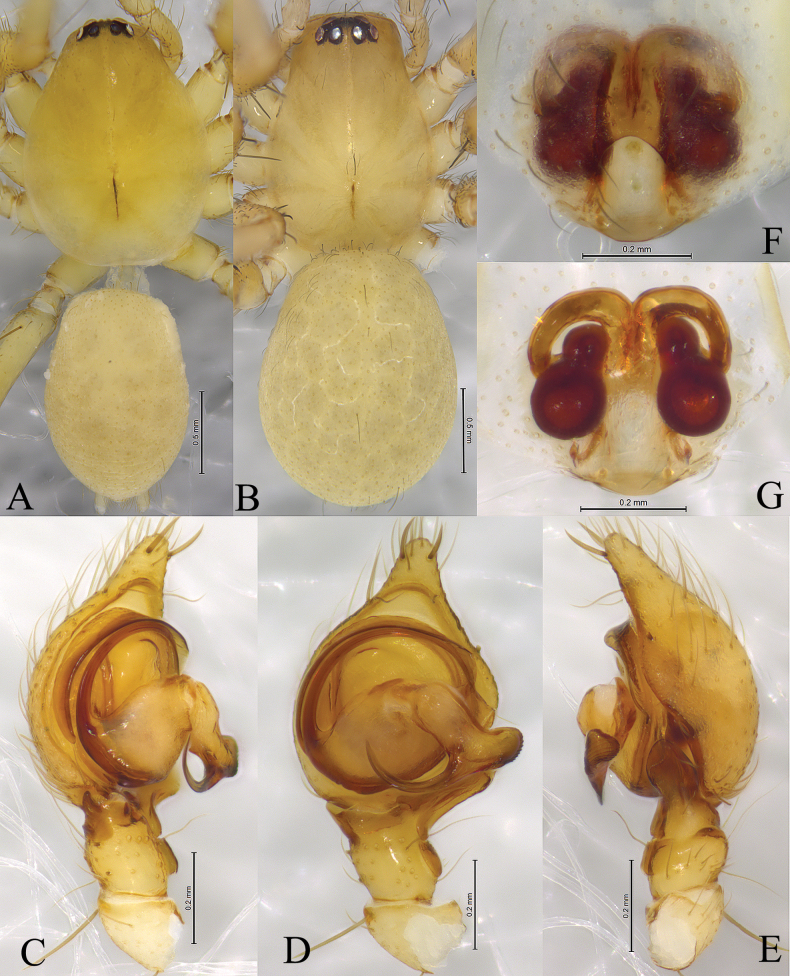
*Cicurinalongihamata* sp. nov. holotype male (**A, C–E**) and paratype female (**B, F, G**) **A** male habitus, dorsal view **B** female habitus, dorsal view **C** left male palp, prolateral view **D** same, ventral view **E** same, retrolateral view **F** epigyne, ventral view **G** vulva, dorsal view.

#### Description.

**Male holotype** (Fig. 4A) total length 3.66. Carapace 1.91 long, 1.43 wide; opisthosoma 1.72 long, 1.22 wide. Eye sizes and interdistances: AME 0.03, ALE 0.10, PME 0.07, PLE, 0.10; AME–AME 0.04, AME–ALE 0.02, PME–PME 0.10, PME–PLE 0.05, ALE–PLE 0.02. MOA 0.19 long, anterior width 0.10, posterior width 0.25. Clypeus height 0.16. Chelicerae with 3 promarginal and 7 retromarginal teeth. Leg measurements: I 4.95 (1.48, 1.73, 0.98, 0.76); II 4.45 (1.36, 1.53, 0.92, 0.64); III 4.07 (1.20, 1.32, 0.95, 0.60); IV 5.49 (1.56, 1.81, 1.34, 0.78). Leg formula: 4123.

***Palp*** (Figs 3A, B, 4C–E). Retrolateral tibial apophysis wide, with a single fold and truncates apex. The base of retrolateral tibial apophysis with two small apophyses, extending ventrally and dorsally. Embolus strong, originating at approximately 9-o’clock position, anterior part resting in the long groove of conductor. Conductor strong, with a beak-like end.

**Female paratypes** (SWUC-T-CI-11-02, Fig. 4B) total length 3.33–4.95 (*N* = 2). One female total length 3.33. Prosoma 1.70 long, 1.15 wide; opisthosoma 1.48 long, 1.20 wide. Eye sizes and interdistances: AME 0.06, ALE 0.11, PME 0.10, PLE, 0.11; AME–AME 0.05, AME–ALE 0.01, PME–PME 0.08, PME–PLE 0.06, ALE–PLE 0.05. MOA 0.25 long, anterior width 0.16, posterior width 0.28. Clypeus height 0.12. Leg measurements: I 4.18 (1.22, 1.542, 0.83, 0.61); II 3.65 (1.08, 1.28, 0.75, 0.54); III 3.29 (0.95, 1.15, 0.69, 0.50); IV 4.44 (1.26, 1.53, 1.05, 0.60). Leg formula: 4123.

***Epigyne*** (Figs 3C, D, 4F, G). Atrium oval. Copulatory openings located anterior of atrium. Copulatory ducts as long as spermathecae diameter, C-shaped. Spermathecae spherical, anteriorly located. Fertilization ducts folded.

**Figure 7.**
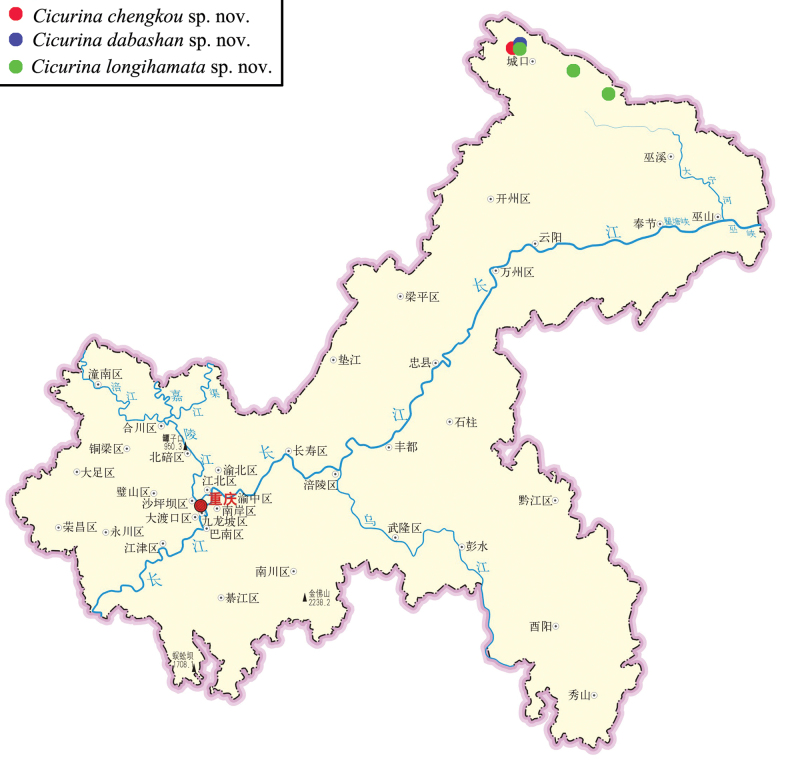
Distribution records of three *Cicurina* species in Chongqing, China.

#### Distribution.

China (Chongqing) (Fig. 7).

### ﻿ Cicurina longihamata sp. nov.

Taxon classificationAnimaliaAraneaeHahniidae

EEB5B56C-A5A5-5AD2-B37D-60F45196CC29

https://zoobank.org/AD3DFE55-EB4B-4BF3-A1B0-85FAF3608780

Figs 5 , 6 , 7 Vernacular name: 长钩洞叶蛛

#### Type material.

***Holotype*** • ♂ (SWUC-T-CI-12-01), **China**, Chongqing City, Chengkou County, Longtian Town, Wuli Village, Daba Mountain National Nature Reserve, 32°05.062'N, 108°38.300'E, elev. 1417 m, 17 September 2012, L.Y. Wang and X.K. Jiang leg. (SWUC). ***Paratypes*** • 6♂ 3♀ (SWUC-T-CI-12-02 to 10), with same data as for holotype • 1♂ 1♀ (SWUC-T-CI-12-11 to 12), Wuli Village, Daba Mountain National Nature Reserve, 32°04.443'N, 108°39.278'E, elev. 1264 m, 16 September 2012, L.Y. Wang and X.K. Jiang leg. • 2♀ (SWUC-T-CI-12-13 to 14), Wuli Village, Daba Mountain National Nature Reserve, 32°04.590'N, 108°39.058'E, elev. 1417 m, 17 September 2012, L.Y. Wang and X.K. Jiang leg. • 1♀ (SWUC-T-CI-12-15), Wuli Village, Daba Mountain National Nature Reserve, 32°03.836'N, 108°40.238'E, elev. 1275 m, 21 March 2013, X.K. Jiang and X.W. Meng leg. • 4♀ (SWUC-T-CI-12-16 to 19), Wuli Village, Daba Mountain National Nature Reserve, 32°03.726'N, 108°40.351'E, elev. 1206 m, 16 March 2018, Z.S. Zhang, L.Y. Wang and Z.S. Wu leg. • 5♀ (SWUC-T-CI-12-20 to 24), Wuli Village, Daba Mountain National Nature Reserve, 32°04.269'N, 108°39.914'E, elev. 1286 m, 16 March 2018, Z.S. Zhang, L.Y. Wang and Z.S. Wu leg. • 3♀ (SWUC-T-CI-12-25 to 27), Heyu Town, Xumu Village, Daba Mountain National Nature Reserve, 31°54.484'N, 109°03.556'E, elev. 1670 m, 27 March 2013, X.K. Jiang and X.W. Meng leg. • 1♀ (SWUC-T-CI-12-28), Xumu Village, Daba Mountain National Nature Reserve, 31°55.393'N, 109°01.930'E, elev. 1593 m, 28 March 2013, X.K. Jiang and X.W. Meng leg. • 1♀ (SWUC-T-CI-12-29), Dongan Township, Chaoyang Village, Daba Mountain National Nature Reserve, 31°47.099'N, 109°14.727'E, elev. 1576 m, 29 March 2013, X.K. Jiang and X.W. Meng leg. • 1♀ (SWUC-T-CI-12-30), Dongan Township, Xingtian Village, Daba Mountain National Nature Reserve, 31°43.426'N, 109°08.563'E, 1391 m, 31 March 2013, X.K. Jiang and X.W. Meng leg.

#### Etymology.

The specific name is a combination of ‘*long*’ and ‘*hamata*’, referring to the long and hook-shaped conductor; adjective.

#### Diagnosis.

The new species is similar to *C.eburnata* Wang, 1994 (Wang et al. 2019: 354, figs 9A–D, 10A–G) in having similar shaped retrolateral tibial apophysis, long and strong embolus, strong and hook-like conductor, posteriorly located epigynal atrium, but differs from the latter by the conductor with a long, acicular and C-shaped end (Figs 5A, B, 6C–E) (vs. short and blunt end in *C.eburnata*), the C-shaped copulatory ducts and gourd-shaped spermathecae (Figs 5D, 6G) (vs. rod-shaped and spherical *C.eburnata*).

#### Description.

**Male holotype** (Fig. 6A) total length 2.93. Prosoma 1.51 long, 1.14 wide; Opisthosoma 1.31 long, 0.86 wide. Eye sizes and interdistances: AME 0.03, ALE 0.09, PME 0.07, PLE, 0.08; AME–AME 0.02, AME–ALE 0.02, PME–PME 0.07, PME–PLE 0.04, ALE–PLE 0.02. MOA 0.14 long, anterior width 0.07, posterior width 0.21. Clypeus height 0.14. Chelicerae with 3 promarginal and 7 retromarginal teeth. Leg measurements: I 3.66 (1.09, 1.31, 0.70, 0.56); II 3.18 (0.94, 1.08, 0.64, 0.52); III 2.85 (0.83, 0.88, 0.69, 0.45); IV 3.99 (1.10, 1.32, 0.95, 0.62). Leg formula: 4123.

***Palp*** (Figs 5A–B, 6C–E). Retrolateral tibial apophysis wide. The base of retrolateral tibial apophysis with two small apophyses, extending ventrally and dorsally. Embolus strong, originating at approximately 9-o’clock position, anterior part resting in the groove of conductor. Conductor strong, with a long and J-like end.

**Female paratype** (SWUC-T-CI-12-02, Fig. 6B) total length 2.94. Prosoma 1.46 long, 0.99 wide; opisthosoma 1.57 long, 1.18 wide. Eye sizes and interdistances: AME 0.04, ALE 0.10, PME 0.07, PLE, 0.09; AME–AME 0.02, AME–ALE 0.02, PME–PME 0.07, PME–PLE 0.04, ALE–PLE 0.02. MOA 0.18 long, anterior width 0.08, posterior width 0.22. Clypeus height 0.12. Leg measurements: I 3.56 (1.10, 1.30, 0.65, 0.51); II 3.16 (0.95, 1.11, 0.62, 0.48); III 2.86 (0.87, 0.90, 0.66, 0.43); IV 3.99 (1.18, 1.36, 0.93, 0.52). Leg formula: 4123.

***Epigyne*** (Figs 5C–D, 5F–G). Atrium oval. Copulatory openings located anterior of atrium. Copulatory ducts strongly curved, circular. Spermathecae large and gourd-shaped. Fertilization ducts long and hook-like.

#### Variation.

Males (*N* = 8) total length 2.80–3.15; females (*N* = 22) total length 2.90–3.49.

#### Distribution.

China (Chongqing) (Fig. 7).

## Supplementary Material

XML Treatment for Cicurina chengkou

XML Treatment for Cicurina dabashan

XML Treatment for Cicurina longihamata

## ﻿Additional information

### Author ORCIDs

Lu-Yu Wang https://orcid.org/0000-0002-5250-3473

Yan-Nan Mu https://orcid.org/0000-0002-2504-673X

Zhi-Sheng Zhang https://orcid.org/0000-0002-9304-1789

### Data availability

All of the data that support the findings of this study are available in the main text.

## References

[B1] Álvarez-Padilla F Hormiga G (2007) A protocol for digesting internal soft tissues and mounting spiders for scanning electron microscopy. Journal of Arachnology 35(3): 538–542. 10.1636/Sh06-55.1

[B2] Deng HP (2015) Biodiversity of Chongqing Daba Mountain National Nature Reserve. Science Press, Beijing, 279 pp.

[B3] Fu L Zhang ZS Zhang F (2016) New *Otacilia* species from Southwest China (Araneae: Phrurolithidae). Zootaxa 4107(2): 197–221. 10.11646/zootaxa.4107.2.4 27394814

[B4] Li SQ Wang XX (2017) New cave-dwelling spiders of the family Dictynidae (Arachnida, Araneae) from Guangxi and Guizhou, China. Zoological Systematics 42(2): 125–228. 10.11865/zs.201711

[B5] Liao RR Yin HQ He AL Xu X (2022) Description of three new species of the genus *Cicurina* Menge, 1871 from Guangdong, China (Araneae, Hahniidae). Zootaxa 5188(5): 477–488. 10.11646/zootaxa.5188.5.4 37044765

[B6] Lin YJ Li SQ Pham DS (2023) Taxonomic notes on some spider species (Arachnida: Araneae) from China and Vietnam. Zoological Systematics 48(1): 1–99. 10.11865/zs.2023101

[B7] Lin YJ Li SQ Mo HL Wang XH (2024) Thirty-eight spider species (Arachnida: Araneae) from China, Indonesia, Japan and Vietnam. Zoological Systematics 49(1): 4–98. 10.11865/zs.2024101

[B8] Mu Y Zhang F (2021) Seven new *Otacilia* Thorell, 1897 species from China (Araneae: Phrurolithidae). Zootaxa 5032(4): 533–548. 10.11646/zootaxa.5032.4.4 34811111

[B9] Wang LY Zhou GC Peng XJ (2019) Four new species of the spider genus *Cicurina* Menge, 1871 from China (Araneae: Dictynidae). Zootaxa 4615(2): 351–364. 10.11646/zootaxa.4615.2.7 31716347

[B10] WSC (2024) World Spider Catalog. Version 25.5. Natural History Museum Bern. [Accessed on: 2024-10-31] 10.24436/2

[B11] Zhang L Wang LY Zhang ZS (2018) The first record of *Amaurobius* C.L. Koch, 1837 (Araneae, Amaurobiidae) from China, with description of two new species. Zootaxa 4402(2): 363–372. 10.11646/zootaxa.4402.2.8 29690272

